# Cochlear Implant Electrode Impedance as Potential Biomarker for Residual Hearing

**DOI:** 10.3389/fneur.2022.886171

**Published:** 2022-06-27

**Authors:** Wilhelm Wimmer, Luca Sclabas, Marco Caversaccio, Stefan Weder

**Affiliations:** ^1^Hearing Research Laboratory, ARTORG Center for Biomedical Engineering Research, University of Bern, Bern, Switzerland; ^2^Department of ENT—Head and Neck Surgery, Inselspital, Bern University Hospital, University of Bern, Bern, Switzerland

**Keywords:** hearing preservation monitoring, cochlear health, impedance telemetry, hearing threshold, electrode impedance, follow-up

## Abstract

**Introduction and Objectives:**

Among cochlear implant candidates, an increasing number of patients are presenting with residual acoustic hearing. To monitor the postoperative course of structural and functional preservation of the cochlea, a reliable objective biomarker would be desirable. Recently, impedance telemetry has gained increasing attention in this field. The aim of this study was to investigate the postoperative course of the residual acoustic hearing and clinical impedance in patients with long electrode arrays and to explore the applicability of impedance telemetry for monitoring residual hearing.

**Methods:**

We retrospectively analyzed records of 42 cochlear implant recipients with residual hearing covering a median postoperative follow-up of 25 months with repeated simultaneous pure tone audiometry and impedance telemetry. We used a linear mixed-effects model to estimate the relation between clinical electrode impedance and residual hearing. Besides the clinical impedance, the follow-up time, side of implantation, gender, and age at implantation were included as fixed effects. An interaction term between impedance and follow-up time, as well as subject-level random intercepts and slopes, were included.

**Results:**

Loss of residual hearing occurred either during surgery or within the first 6 post-operative months. Electrode contacts inserted further apically (i.e., deeper) had higher impedances, independent of residual hearing. The highest impedances were measured 1 month postoperatively and gradually decreased over time. Basal electrodes were more likely to maintain higher impedance. Follow-up time was significantly associated with residual hearing. Regardless of the time, we found that a 1 kΩ increase in clinical impedance was associated with a 4.4 dB deterioration of residual hearing (*p* < 0.001).

**Conclusion:**

Pure tone audiometry is the current gold standard for monitoring postoperative residual hearing. However, the association of clinical impedances with residual hearing thresholds found in our study could potentially be exploited for objective monitoring using impedance telemetry. Further analysis including near-field related impedance components could be performed for improved specificity to local immune responses.

## 1. Introduction

An increasing proportion of patients with residual hearing are being considered (CI) candidates today. After implantation, patients with successfully preserved hearing can perform better in complex listening environments (i.e., speech comprehension in noise), perceive music more naturally, and have improved spatial perception (1–3). Approximately 50% of CI recipients retain their residual hearing for several years (4, 5). Some of these patients meet the criteria for electro-acoustic stimulation (EAS). In these cases, the ear can be stimulated both acoustically (*via* the hearing aid) and electrically (*via* the implant), resulting in even better speech understanding (6, 7). In case of unsuccessful hearing preservation, intraoperative loss or residual hearing is thought to be the result of traumatic events during the insertion and implantation of the electrodes (8–11). Postoperative hearing loss, on the other hand, is suspected to be caused by an immune reaction to the electrode array (12), intracochlear inflammatory responses, e.g., to blood components entering the inner ear (13), intracochlear scar tissue formation (14), or a progression of hearing loss independent of the implanted device (1).

To better understand the postoperative course of inner ear function and to enable frequent monitoring, an objective intracochlear biomarker would be most appropriate. Impedance telemetry could be practicable and recently gained attention in this context (15, 16). The assessment of impedances is easy and quickly performed, does not require the active participation of the patient, is independent of the middle ear status (e.g., also feasible in case of a haematotympanum), and can be performed telemetrically or even through telemedical access (17).

Sudden increases in postoperative impedance values (so-called “impedance spikes”) were linked with inner-ear events leading to loss of residual hearing and vertigo (18, 19). In an animal model, Bester et al. (8) reported higher impedances in the presence of blood clots around the electrode, which could initiate an inflammatory response that affects the residual function of the inner ear. However, despite the promising indication, impedance telemetry remains a non-established monitoring tool. Konrad et al. (20) performed an analysis in patients implanted with short electrode arrays and found a correlation between impedance and threshold changes, but it was too inconsistent to imply a true relationship. In the context of longer electrode arrays, whose impedance has been shown to depend on insertion depth (16), we wanted to investigate whether a relationship could exist between clinical impedance and residual hearing in the long term. Therefore, the aim of this study was to extend the analysis to CI-models with deeper inserted electrode arrays. We investigated the postoperative courses of (i) residual acoustic hearing, (ii) clinical impedance, and (iii) we evaluated the potential of impedance telemetry for residual hearing monitoring by assessing the relation between both factors.

## 2. Materials and Methods

### 2.1. Study Design and Subjects

This retrospective study was conducted with the approval of the local institutional review board (approval number: Basec ID 2020-02978). We reviewed all adult patients who received a CI at our tertiary referral hospital between January 2009 and June 2021 (*N* = 704). Of these, only cases were included, who (i) received a MED-EL (Innsbruck, Austria) implant, (ii) had a measurable residual hearing of at least 5 dB between 125 and 1,000 Hz in the preoperative pure tone audiogram, and (iii) had a well-documented postoperative follow-up with two or more pure tone audiograms and concurrent impedance telemetry for at least 6 months.

### 2.2. Audiometric Assessment and Residual Hearing

**Figure 1:**
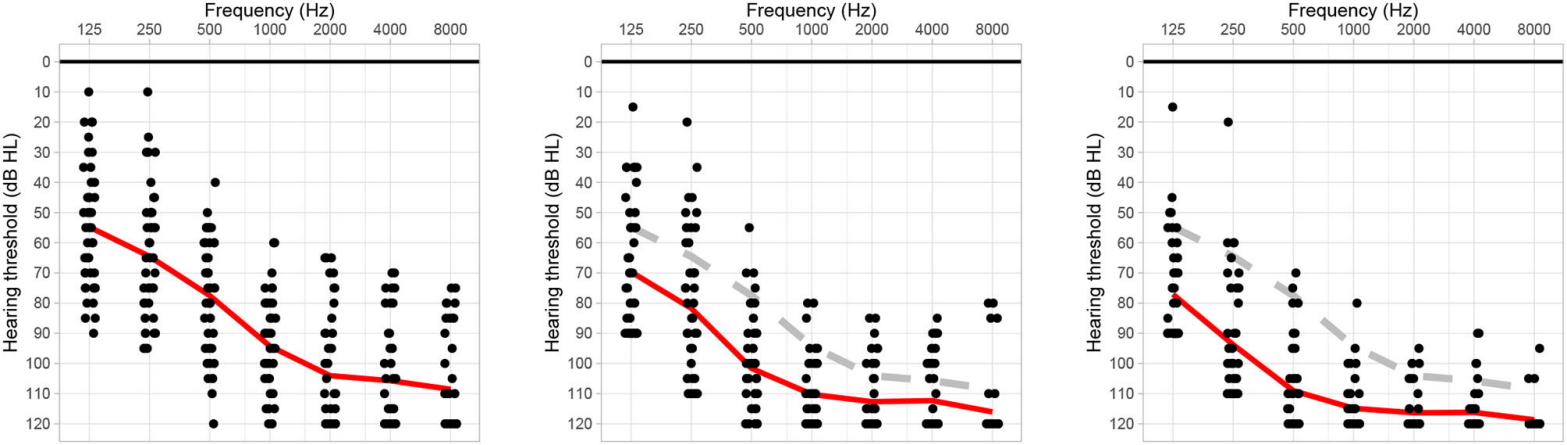
Preoperative, 3-month postoperative, and 6-months or the next later follow-up session air conduction hearing thresholds (left, center, and right audiograms, respectively). The red line indicates the average hearing thresholds, the gray dashed line shows the preoperative reference.

All audiological assessments were performed in routine practice in an acoustic chamber with a clinical audiometer. Pure tone air conduction hearing thresholds were measured in dB hearing level (HL) at 125, 250, 500, 1,000, 2,000, 4,000, and 8,000 Hz using either headphone or insert earphones. Figure 1 summarizes the preoperative, 3-, and 6-month postoperative air conduction hearing thresholds of the subjects. The residual hearing was defined in absolute values as the difference between the maximum detectable levels (i.e., 90 dB HL at 125 Hz, 110 dB HL at 250 Hz, and 120 dB HL for the other frequencies) and the hearing thresholds. In addition to the absolute PTA, we computed relative values of residual hearing according to (21).

### 2.3. Electrode Impedances

Impedance telemetry was performed during the same sessions as pure tone audiometry, according to the clinical standard procedure at our institution. For the analysis, we exported the clinical impedance (in kΩ) from the manufacturer's telemetry software (Maestro, MED-EL). The clinical electrode impedance includes near-field interface impedance and far-field tissue resistance and can be found from the diagonal elements of the measured voltage matrix (16). In addition, to analyze impedance progression over time for all subjects, the clinical impedance values were extracted at defined intervals according to clinical routine, i.e., preoperatively (month 0), first activation (month 1), and consecutive fitting sessions (months 3, 6, 12, and 24). Extracochlear electrodes, as indicated by surgical and radiological reports, were excluded from the analysis (refer to Table 1).

**Table 1:** Subject demographics and functional outcomes, sorted by relative preservation of residual hearing after 6 months. F, female; M, male; R, right; L, left.

**ID**	**Gender**	**Age**	**Side**	**Etiology**	**Follow-up**	**Electrode array**	**Insertion**	Word recognition scores^*^		Hearing preservation^**^		
		**(years)**			**(months)**			**Monosyllables**	**Numbers**	**Absolute**	**Relative**	**Category**
								**(%)**	**(%)**	**(dB HL)**	**(%)**	
5	F	57	R	Progressive	49	FLEX^24^	Partial (11 of 12)	100	100	−5	86	*1*
36	M	60	R	Progressive	41	FLEX^24^	Full	40	90	−4	83	*1*
18	F	56	L	Progressive	15	FLEX^28^	Full	50	100	−8	82	*1*
15	M	69	L	Progressive	47	FLEX^24^	Full	70	100	−13	80	*1*
11	F	61	R	Progressive	6	FLEX^24^	Full	50	90	−15	65	*2*
23	F	67	L	Progressive	58	FLEX^28^	Full	65	100	−20	65	*2*
2	F	55	L	Progressive	84	FLEX^24^	Full	95	100	−19	63	*2*
42	F	53	R	Progressive	94	FLEX^24^	Full	100	100	−23	61	*2*
16	F	59	L	N/A	28	FLEX^28^	Full	85	100	−24	59	*2*
40	F	24	L	Progressive	13	FLEX^28^	Full	45	100	-10	59	*2*
17	M	70	R	Progressive	14	FLEX^28^	Full	80	100	−14	58	*2*
26	F	26	L	Progressive	49	FLEX^28^	Full	95	100	−8	56	*2*
6	F	43	R	Progressive	56	FLEX^24^	Full	10	100	−10	56	*2*
30	F	39	L	Progressive	8	FLEX^24^	Full	75	100	−13	55	*2*
21	F	80	R	Progressive	13	FLEX^28^	Full	40	100	−16	55	*2*
13	M	74	L	Sudden	13	FLEX^28^	Partial (11 of 12)	100	100	−17	48	*2*
8	M	60	R	Progressive	13	FLEX^24^	Full	65	100	−31	48	*2*
28	F	43	L	Progressive	25	FLEX^28^	Full	40	40	−19	40	*2*
33	F	52	R	Progressive	41	FLEX^24^	Partial (9 of 12)	70	90	−41	31	*2*
41	F	73	R	Hydrops	22	FLEX^28^	Full	20	80	−19	25	*2*
31	F	32	R	Progressive	21	FLEX^28^	Partial (11 of 12)	45	80	-6	23	*3*
37	F	40	L	Progressive	13	FLEX^28^	Full	45	80	−25	23	*3*
32	F	39	R	Progressive	7	FLEX^Soft^	Full	75	100	−14	22	*3*
25	M	26	L	Progressive	104	FLEX^24^	Full	85	90	−49	16	*3*
10	F	61	R	Progressive	42	FLEX^28^	Full	65	100	−41	15	*3*
7	M	71	L	Progressive	88	FLEX^24^	Full	25	100	−43	13	*3*
24	F	62	L	Progressive	31	FLEX^Soft^	Full	65	100	−29	10	*3*
12	F	70	R	Sudden	39	FLEX^28^	Partial (9 of 12)	50	80	−48	10	*3*
29	F	44	R	Progressive	15	FLEX^28^	Full	40	60	−30	9	*3*
35	M	69	R	Progressive	42	FLEX^28^	Full	85	100	−19	5	*3*
1	M	61	R	Progressive	10	FLEX^28^	Full	45	90	−34	5	*3*
20	M	63	R	Sudden	20	FLEX^28^	Full	25	90	−50	3	*3*
4	M	71	L	Progressive	52	FLEX^28^	Full	20	40	−49	2	*3*
39	M	45	L	Progressive	48	FLEX^28^	Full	0	20	−19	1	*3*
34	M	52	L	Trauma	35	Standard	Full	80	100	−10	0	*4*
22	M	64	R	Hydrops	13	FLEX^Soft^	Full	75	100	−33	0	*4*
38	M	42	R	Trauma	6	FLEX^28^	Full	70	100	−9	0	*4*
9	M	46	R	Progressive	25	FLEX^28^	Full	70	100	−38	0	*4*
3	M	59	L	Progressive	13	FLEX^28^	Full	50	100	−15	0	*4*
27	F	35	L	Progressive	42	FLEX^28^	Partial (9 of 12)	50	100	−19	0	*4*
19	M	52	L	Hydrops	13	FLEX^28^	Partial (10 of 12)	20	50	−39	0	*4*
14	F	61	L	Meningitis	7	FLEX^28^	Partial (11 of 12)	0	60	−23	0	*4*

* *German Freiburg monosyllabic word lists at 65 dB sound pressure level (SPL) and German Freiburg numbers at 60 dB SPL after 6 months.*

** *Residual hearing category according to (21) after 6 months*.

### 2.4. Statistical Analysis

Descriptive statistics were used to summarize demographic data and functional outcomes. We used linear mixed-effects models to assess the relation between CI electrode impedance and residual hearing over time. We calculated separate models accounting for the impedance of all active electrodes as well as only the apical (contacts 1–4), middle (contacts 5–8), and basal electrodes (contacts 9–12). The models employed residual hearing (in dB HL) as the dependent variable and electrode impedance (in kΩ) as the independent variable. The follow-up time (in months), side of implantation, gender, and age were additionally included as fixed effect variables. An interaction term between impedance and follow-up time was also included to account for time-dependent variations of the impedance. Subject-level random intercepts were used to account for repeated measurements. Random slopes of impedance were included to enable varying effects for individual subjects. Using the random slope mixed-effects model, subjects may have individual intercepts (random intercept) and individual slopes (i.e., differently strong effects; random slope) around common effects in the association between residual hearing and impedance. We used the *R environment* (v4.0.3) and the *lme4* package (v1.1) for the statistical analysis (22). Audiograms were generated using the *audiometry* package (v0.3.).

## 3. Results

### 3.1. Demographics

Of the 704 entries in our database, 119 patients had a post-operative audiogram available and measurable residual hearing of at least 5 dB between 125 and 1,000 Hz. Among these, 79 were implanted with a MED-EL device. Forty-two patients had two or more pure tone audiograms with concurrent impedance telemetry for at least 6 months and were included in our analysis (21 men and 21 women, mean age at implantation of 54 years). Most commonly, CIs with Flex^28^ electrode arrays were implanted (*N* = 26). Eight patients had a partial electrode insertion, with up to three extracochlear electrodes. Six months post activation, the median word recognition score in quiet was 58% (interquartile range [40, 73%]) for the German Freiburg monosyllabic word test and 100% (interquartile range [90, 100%]) for the German Freiburg numbers test. Patients had a median follow-up over 25 months (interquartile range [13 months, 46 months]) with pure tone audiograms and simultaneous impedance telemetry.

### 3.2. Residual Hearing Progression Over Time

**Figure 2:**
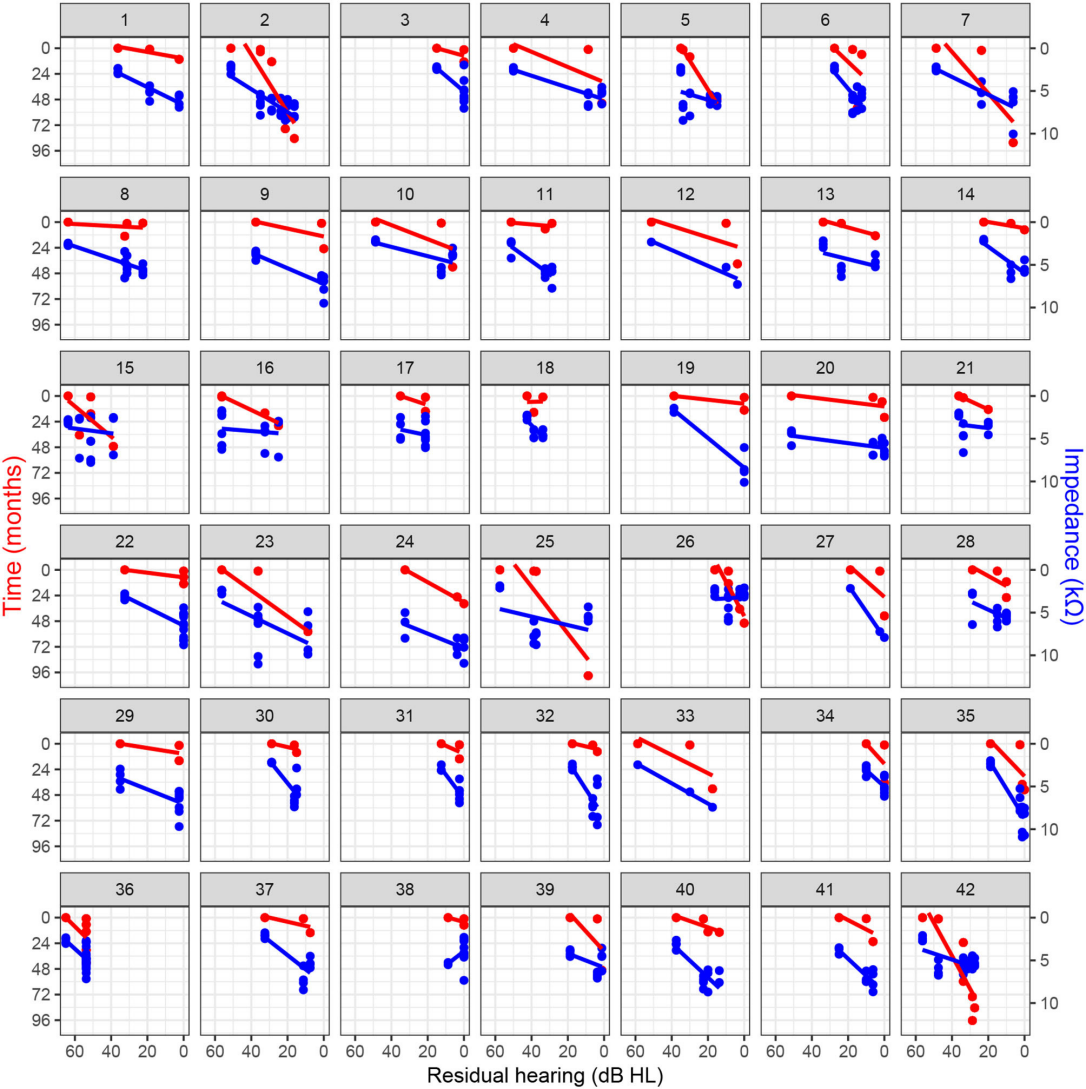
Residual hearing progression over time and association with clinical electrode impedance for basal electrodes (i.e., contacts 9–12) with regression lines.

One month post-operatively, seven patients had *complete hearing preservation* (category 1), 20 patients had *partial hearing preservation* (category 2), 10 patients presented *minimal hearing preservation* (category 3), and five patients had *complete loss of residual hearing* (category 4). Most commonly, the transition from categories 1–2 occurred within the first 3 months after surgery. On average, cochlear implantation worsened the unaided hearing thresholds by 13.6 dB HL after 3 months and by 19.5 dB HL after 6 months (Figure 1). The categorization of hearing preservation after 6 months according to (21) is shown in Table 1. The individual course of residual hearing over time is illustrated in Figure 2.

In general, all linear mixed-effects models used (i.e., models including all electrodes, or only basal, middle, and apical electrodes) showed comparable results and significance. The most prominent effects, which are presented herein, were observed for the model including basal electrodes (i.e., 9–12). The results for the additional models including either all, middle, or apical electrodes are summarized in the Supplementary Material. No dependence of gender and implantation side on residual hearing was found (Table 2). For the model including all electrodes, age at implantation showed a small association with decreased residual hearing (refer to Supplementary Material). The follow-up time after implantation was related to a statistically significant decrease in residual hearing (−0.7 dB HL per month or 8.4 dB HL per year; *p* < 0.001).

**Table 2:** Linear mixed-effects model summary table for residual hearing (in dB HL) including basal electrodes (i.e., 9–12).

	**Coefficient**	**95% CI**	***p*-value**
*Intercept*	56.5	[39.9, 73.1]	<0.001
Time (months)	−0.7	[−0.8, −0.6]	<0.001
Impedance (kΩ)	−4.4	[−5.3, −3.5]	<0.001
Interaction of time with impedance	0.08	[0.06, 0.09]	<0.001
Side	0.4	[-9.0,9.8]	0.94
Gender	−0.4	[−2.3, 1.4]	0.64
Age at implantation (years)	−0.2	[−0.5, 0.1]	0.18

### 3.3. Clinical Impedance Progression Over Time

**Figure 3:**
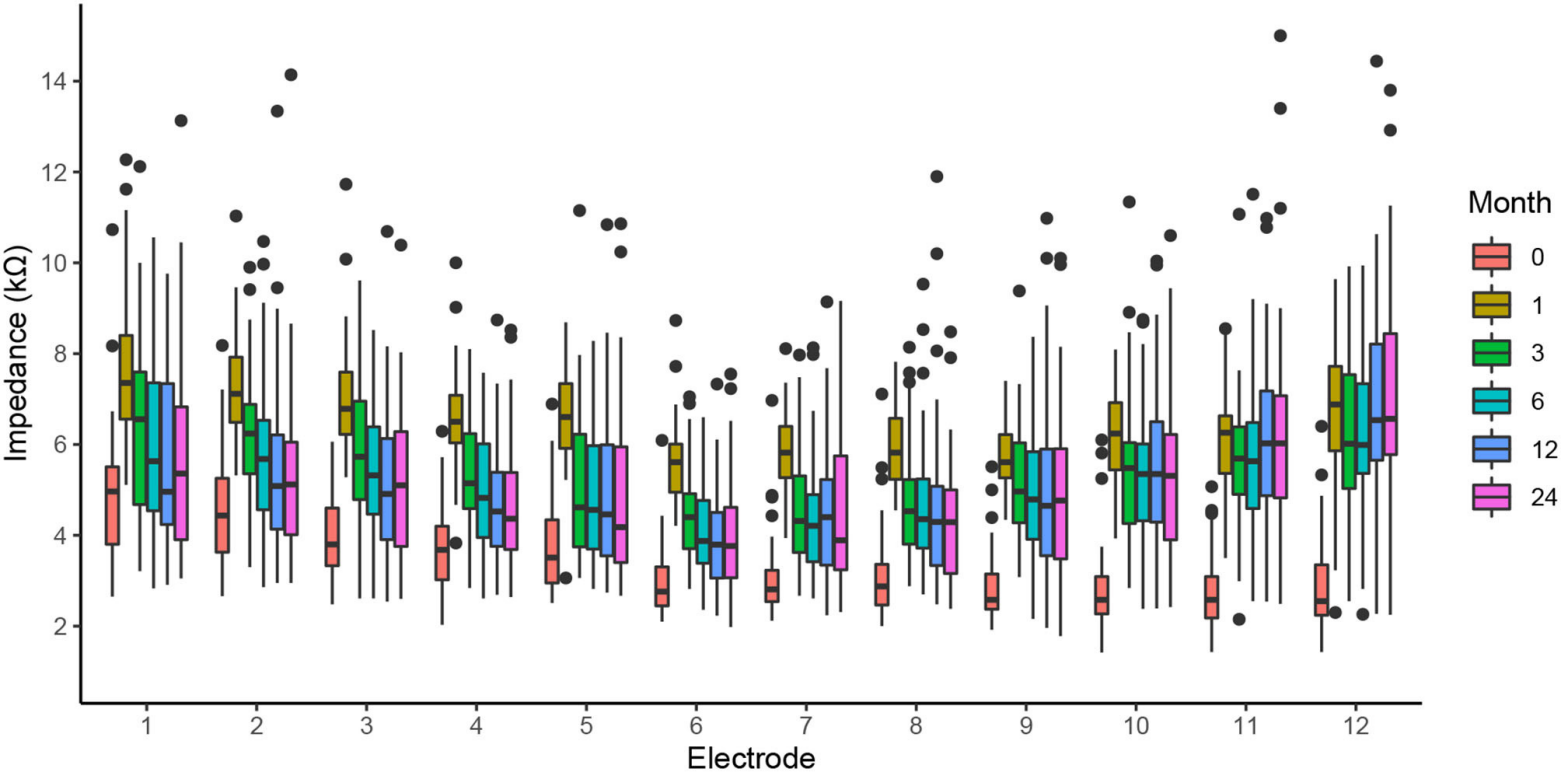
The clinical impedance of all electrodes for the intraoperative measurement (month 0), first activation session (month 1), and follow-up fitting sessions (months 3–24).

We found no dependence of the electrode impedance on gender, age, or implantation side. However, there was a time dependence of impedance, with the lowest values observable immediately after the insertion (month 0; Figure 3). Impedances were highest at the first activation session, i.e., 1 month post-operatively. After this peak, the impedances of apical electrodes decreased toward the intraoperative values, whereas the basal electrodes stabilized at higher levels, resulting in a U-shaped distribution. Finally, the dispersion of impedance values was smallest for intraoperative measurement (month 0), with an average interquartile range of 1.2 kΩ. At successive measurement points, the distribution of impedance values gradually increased, with an average interquartile range of 1.3 and 1.9 kΩ at the 1 and 24 month sessions, respectively. During the intraoperative measurements, the direct influence of the electrode insertion depth on impedances can be observed (16). On average, intraoperatively, the highest impedances occurred on apical contacts (4.3 kΩ; electrodes 1–4), monotonically decreasing toward the middle (3.2 kΩ; electrodes 5–8) and basal contacts (2.9 kΩ average for electrodes 9–12).

**Figure 4:**
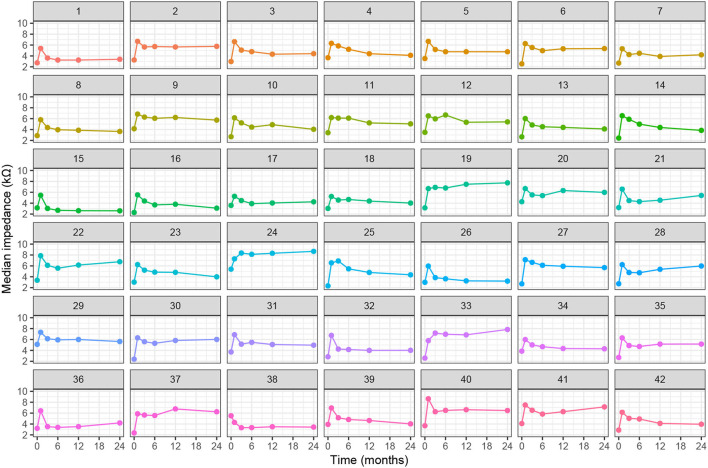
Individual progression of clinical electrode impedances (median value of all electrodes) over time.

Figure 4 shows the individual, postoperative courses of clinical impedance expressed as the median value over all electrodes. Insertion of the electrode array leads to a steep average increase in electrode impedance by 3.1 kΩ, range (1.2–5.0 kΩ), visible for all patients. Subsequently and in most cases, impedances decreased again and, compared to intraoperative measurements, stabilized at a higher level. In four cases, there was a long-term impedance increase beyond the 1-month peak (subjects 19, 24, 33, and 37). In our cohort, a prominent spike response in median impedances was only found in subject 12 after 6 months.

### 3.4. Association of Residual Hearing and Impedance

Loss of residual hearing was significantly correlated with impedance changes on basal electrodes, independently of the follow-up time (Table 2). The association was also observed in the other linear mixed-effects models (including all electrodes, only middle, or only apical electrodes; refer to Supplementary Material). Figure 2 illustrates the relationship between impedance and residual hearing for individual subjects. The majority of subjects expose a negative association (i.e., the slope of the change) between impedance and residual hearing. The association is less pronounced in subjects 15, 16, 17, 21, 26, and 36, and anti-correlated in subject 38. Patients, who maintained lower impedances over time, were more likely to preserve residual hearing. In contrast, higher impedance values were related to residual hearing loss, with an impedance increase of 1 kΩ being associated with a loss of residual hearing of 4.4 dB HL (*p* < 0.001).

## 4. Discussion

Pure tone audiometry is state-of-the-art when assessing residual hearing. As a subjective, behavioral measurement method, it requires the active participation of the patient and inconsistent responses can occur. In contrast, objective measures could provide additional information independent of the patient's behavior. This attribute would be desirable for postoperative monitoring of the residual inner ear function. Thereby, impedance telemetry could be a promising objective measurement tool, as it can be performed with little effort and can even be self-initiated by the patient. Furthermore, impedance measurements could still be informative in patients with absent residual hearing, e.g., impedance spikes as a surrogate marker of inner-ear events (18).

In our study sample, 64% of the study participants had full or partial hearing preservation at implant activation, 1 month after surgery. This number was further decreased to 48% at the 6-month follow-up. Consequently, most losses of residual hearing occurred intraoperatively or in the early postoperative phase. Reasons can be a traumatic electrode insertion (9, 23, 24) or an inflammatory response with resulting scar tissue formation (12–14). In addition, the presence of the electrode array can influence the mechanical properties of the cochlea and, thus, residual hearing thresholds (25, 26). Our results are in line with previously published literature (1, 27). In general, there was a dependence of residual hearing on both age and follow-up time after implantation. The average reduction of 0.5 dB HL per year of age at surgery (only statistically significant for the model with all electrodes) could be explained by age-related hearing loss, whereas we attribute the decrease in low frequency hearing of 8.4 dB HL per year of postoperative follow-up to the progression of foreign body reaction, inflammation, and wound healing (1, 28).

### 4.1. Development of Impedance Values Over Time

Our measurements showed a noticeable time dependence of the impedance values. Intraoperatively, impedances were lowest; 1 month after implantation, the impedances reached peak values and subsequently decreased in most cases. Parreño et al. (17) showed with daily measurements that maximum values are reached after 18 days. A similar pattern with a peak after a few weeks and subsequent stabilization at a lower level can be observed in the humoral immune response to infection processes (29). Since the implanted electrode acts like a foreign body (12, 13), it can be speculated that the measured impedance values may reflect this immune response.

Besides follow-up time, the position of the electrode inside the cochlea has a substantial influence on impedance values. In the intraoperative measurements, impedances decreased from apical toward basal electrode positions. This is a well-known finding, that can even be exploited to estimate the linear and angular insertion depth of the electrode contacts (16) and potentially at later postoperative stages. Over time, impedances of basal electrodes remained high, whereas impedances of apical electrodes decreased again, ultimately, leading to a long-term observable U-shaped distribution of impedances. This is an interesting observation that was not specifically made in patients with shorter electrodes (19, 20). We hypothesize that these basal impedance increases could indicate post-operative tissue changes within the cochlea.

With regard to impedance spikes, in our cohort, only one noteworthy event could be observed in our data (subject 12 after 6 months). According to the medical record, this impedance spike was not related to a clinical symptom (i.e., hearing loss, vertigo, or tinnitus). However, most often, the previously described phenomenon usually occurs at a later stage after implantation and only in a subset of patients (according to the authors, in 17%) (18).

### 4.2. Correlation of Residual Hearing and Impedance

We could observe a significant association between the course of residual hearing and impedance values. The highest impedance values and at the same time the most frequent loss of acoustic hearing was in the first three months after implantation. Jia et al. (30) demonstrated that the surgical technique influences postoperative impedances. Impedance increases are probably caused by scar formation in the inner ear, which also leads to a deterioration of residual cochlear function (28, 31). Heutink et al. (32) showed in ultra-high-resolution computed tomography images that impedance changes are associated with new bone formation in the inner ear. However, ossification was observed mainly in the basal part of the cochlea. Residual hearing, on the other hand, is more likely to be found in the low frequency range and, thus, in more apical cochlear regions. Nevertheless, the mechanical properties and the tissue present in the basal part of the cochlea might influence the residual hearing thresholds in the more apical regions of the cochlea.

### 4.3. Limitations

Our study population is too small to draw general conclusions. The choice of a linear model represents a simplification of the more complex hidden processes caused by implantation. In particular, we expect the postoperative short-term responses to exhibit nonlinear behavior. However, in the longer term, individual associations, as shown in Figure 2, suggest that a linear model seems reasonable and an allows intuitive interpretation of the results given the exploratory nature of this study. In this context, a further limitation is that we considered only clinical electrode impedances. Analysis of near-field related subcomponents of the clinical impedance, which can be estimated from the voltage matrix (16), could enable higher specificity in the estimation of local tissue characteristics (33, 34). Depending on the implant manufacturer, impedance telemetry is performed differently and may include contributions of the polarization impedance. For these reasons, our findings need to be confirmed with larger sample sizes, including implant systems of other manufacturers.

## 5. Conclusion

The pure tone audiogram remains the gold standard for measuring postoperative residual hearing. Additional objective bio-markers would be desirable to complement these measurements with information about the inner ear independent of the patients' performance. In our study sample, we found an association of residual hearing with clinical impedances indicating its potential use. As this study is exploratory, our results need to be confirmed in larger samples. Analysis of near-field related subcomponents of the electrode impedance could further improve the applicability of impedance telemetry for residual hearing monitoring.

## Data Availability Statement

The original contributions presented in the study are included in the article/Supplementary Material, further inquiries can be directed to the corresponding author/s.

## Ethics Statement

The studies involving human participants were reviewed and approved by Kantonale Ethikbehörde Bern. The patients/participants provided their written informed consent to participate in this study.

## Author Contributions

WW: conceptualization, methodology, formal analysis, and writing—original draft. LS: investigation and data curation. MC: supervision, project administration, and funding acquisition. SW: conceptualization, methodology, project administration, and writing—original draft. All authors contributed to the article and approved the submitted version.

## Funding

This study was funded by the ENT Department, Bern University Hospital. Open access funding provided by University of Bern.

## Conflict of Interest

The authors declare that the research was conducted in the absence of any commercial or financial relationships that could be construed as a potential conflict of interest.

## Publisher's Note

All claims expressed in this article are solely those of the authors and do not necessarily represent those of their affiliated organizations, or those of the publisher, the editors and the reviewers. Any product that may be evaluated in this article, or claim that may be made by its manufacturer, is not guaranteed or endorsed by the publisher.
