# Supplementary material for: Cochlear Implant Electrode Impedance as Potential Biomarker for Residual Hearing
Source: Front Neurol. 2022 Jun 27;13:886171. doi: 10.3389/fneur.2022.886171 (PMC9271767; doi:10.3389/fneur.2022.886171)
Supplement: Supplementary file 1 [file Data_Sheet_1.PDF]

Supplementary Material

1 ALL ELECTRODES

Table S1. Linear mixed-effects model summary table for residual hearing (in dB HL) including all electrodes (i.e., 1 to 12).

	Coefficient	95% CI	p-value
Intercept	69.4	[53.4,84.9]	<.001
Time (months)	-0.6	[-0.7,-0.6]	<.001
Impedance (kΩ)	-3.7	[-4.4,-3.0]	<.001
Interaction of time with impedance	0.06	[0.05,0.07]	<.001
Side	2.1	[-8.4,12.7]	.70
Gender	-1.2	[-2.3,0.01]	.05
Age at implantation (years)	-0.5	[-0.7,-0.2]	<.001

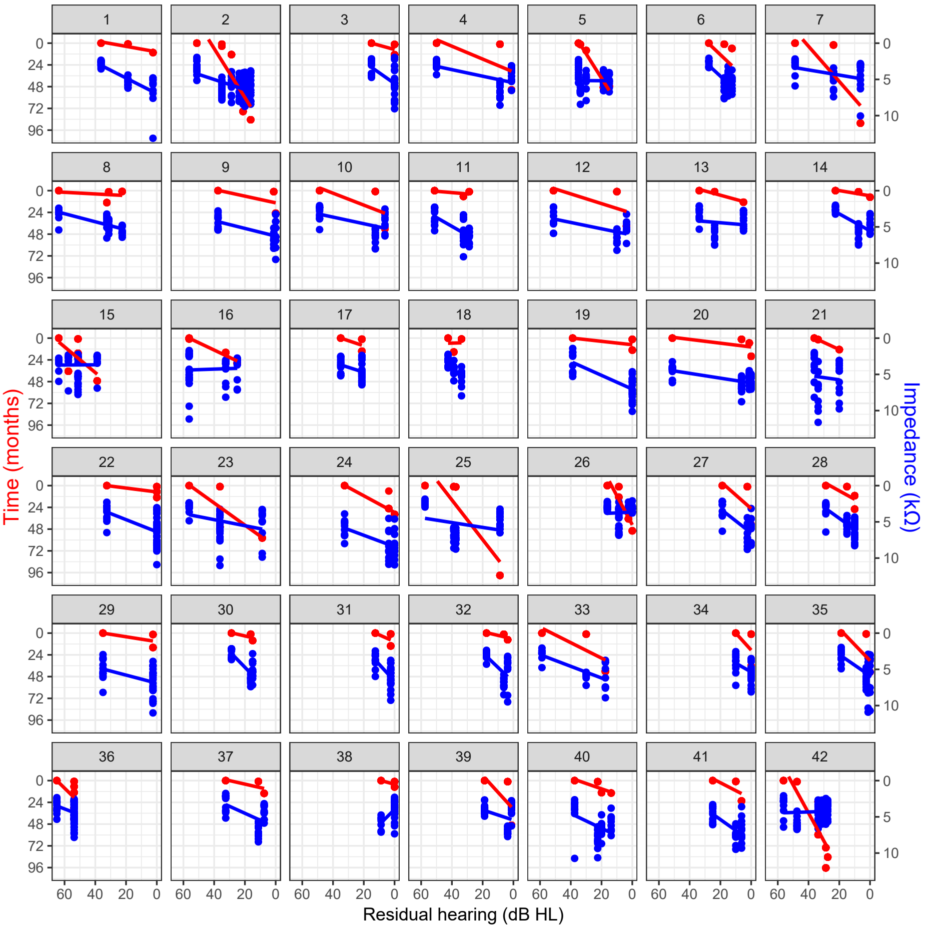

Figure S1. Residual hearing progression over time and association with clinical electrode impedance for all electrodes (i.e., 1 to 12) with regression lines.

2 APICAL ELECTRODES

Table S2. Linear mixed-effects model summary table for residual hearing (in dB HL) including apical electrodes (i.e., 1 to 4).

	Coefficient	95% CI	p-value
Intercept	47.1	[30.3, 64.3]	<.001
Time (months)	-0.6	[-0.7,-0.5]	<.001
Impedance (kΩ)	-4.1	[-5.0,-3.3]	<.001
Interaction of time with impedance	0.04	[0.01,0.06]	.002
Side	0.3	[-8.8,9.5]	.95
Gender	-0.6	[-2.7,1.6]	.60
Age at implantation (years)	-0.03	[-0.3,0.3]	.81

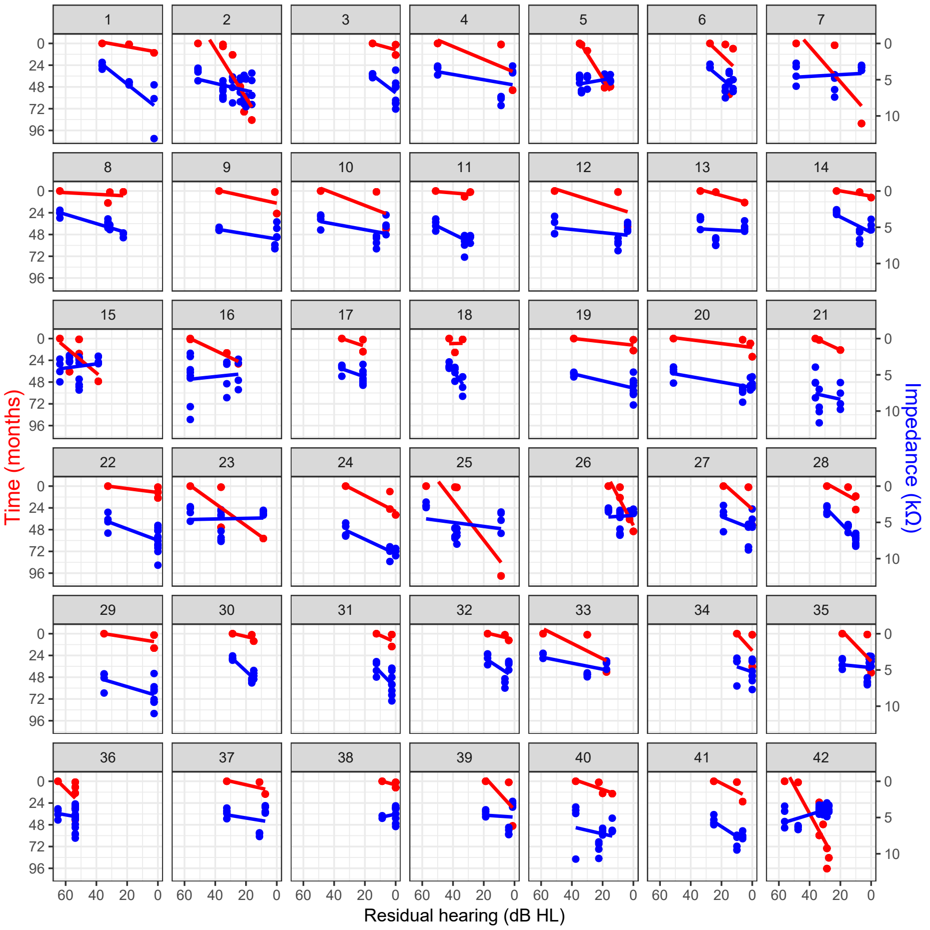

Figure S2. Residual hearing progression over time and association with clinical electrode impedance for apical electrodes (i.e., 1 to 4) with regression lines.

3 MIDDLE ELECTRODES

Table S3. Linear mixed-effects model summary table for residual hearing (in dB HL) including middle electrodes (i.e., 5 to 8).

	Coefficient	95% CI	p-value
Intercept	52.8	[34.9,71.0]	<.001
Time (months)	-0.6	[-0.7,-0.5]	<.001
Impedance (kΩ)	-4.4	[-5.4,-3.4]	<.001
Interaction of time with impedance	0.06	[0.03,0.08]	<.001
Side	-0.2	[-8.9,9.4]	.96
Gender	-2.2	[-4.3,-0.2]	.03
Age at implantation (years)	-0.1	[-0.4,0.2]	.44

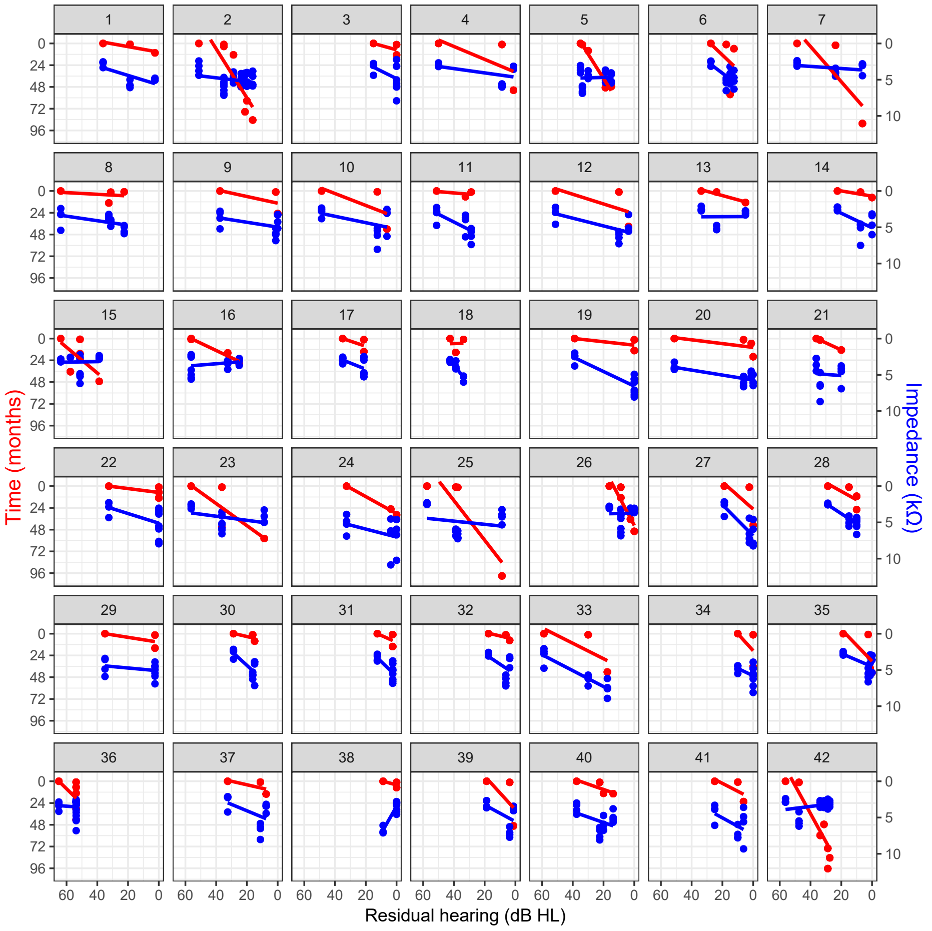

Figure S3. Residual hearing progression over time and association with clinical electrode impedance for middle electrodes (i.e., 5 to 8) with regression lines.

4 BASAL ELECTRODES

Linear mixed-effects model coefficients and the figure for basal electrodes (i.e., 9 to 12) are included in the main publication.
